# Procrastination among US college students: Prevalence and impact relative to common health and life issues

**DOI:** 10.1371/journal.pone.0357714

**Published:** 2026-10-07

**Authors:** Itamar Shatz

**Affiliations:** Department of Theoretical and Applied Linguistics, University of Cambridge, Cambridge, United Kingdom; University of Padova, ITALY

## Abstract

Procrastination is unnecessary delay that can cause various problems. There are uncertainties around how common exactly it is among students, how much it affects them, and how its prevalence and impact compare to those of other issues that students regularly face. Accordingly, this study examines data from 65,950 students (in all degree types and stages), who were surveyed across 97 US institutions of higher education as part of the American College Health Association-National College Health Assessment (ACHA-NCHA). Students were asked about 36 life and health issues, like procrastination, career, finances, appearance, family, bullying, anxiety, depression, ADHD, sleep difficulties, allergies, and influenza. Procrastination was found to be the most common issue that students reported (functionally tied with stress), affecting 72.3% of them. Procrastination was also the issue that most often harmed students’ academic performance, affecting 45.4% of them (62.7% of those who experienced it), which was several times as often as issues like finances (2.8x) and ADHD (3.5x). And, procrastination was the most common cause of significant distress compared to the other life issues, affecting 45.7% of students (63.2% of those who experienced it). These rates were higher among undergraduate students, although procrastination was a leading issue among graduate and professional students too. This sheds light on the scale of student procrastination—both in absolute terms and relative to other issues they face—and highlights the need to address it using relevant interventions.

## Introduction

*Procrastination* is unnecessary delay, which people engage in irrationally, despite it being expected to cause them problems like lower productivity, worse financial situation, worse emotional wellbeing, and worse mental and physical health [1–8]. *Academic procrastination* is a common subset of this, which occurs when students procrastinate on academic tasks like doing homework, writing essays, conducting research, and studying for tests. This behavior can cause similar issues as general procrastination, like stress, as well as academic issues, like getting worse grades, failing assignments, having to repeat courses, and even having to drop out entirely [9–17].

Both general and academic procrastination affect students across the world, who are studying at all levels (including elementary school, middle school, high school, college, and graduate school), are studying all topics (like writing and statistics), are traditional or non-traditional, and are low- or high-achieving [1,11,12,18–20]. It is so common among students that the tendency to delay until right before deadlines—which is highly characteristic of procrastinators [21–24]—is sometimes colloquially called the *student syndrome*. Specifically, past research estimates that while approximately 20% of the general adult population procrastinates chronically, approximately 50% of college students do it, and furthermore, approximately 75% consider themselves procrastinators, and approximately 80%–95% procrastinate to some degree [1,3,25].

Past estimates of the scale of procrastination highlight both the importance of understanding this phenomenon and the need for additional research on it. Specifically, such research could reduce our uncertainty regarding the prevalence of procrastination, which mainly stems from some of its key estimates being based on limited samples. This is the case, for example, for the aforementioned statistic that approximately 75% of students consider themselves procrastinators, which is based on data from just 95 undergraduate students in one university, surveyed around four decades ago [25]. In addition, research on procrastination could complement quantitative estimates of its prevalence with estimates of its impact on measures such as academic performance, and contextualize its prevalence and impact against those of other issues that students face, to clarify the true scale of procrastination, in both absolute and relative terms. Accordingly, the present study aims to answer the following research questions, using a sample that is large, diverse, and recent:

*RQ 1a*- How common is procrastination among students?

*RQ 1b*- How does the prevalence of procrastination compare to the prevalence of other issues that students face?

*RQ 2a*- How often does procrastination negatively impact students’ academic performance?

*RQ 2b*- How does the negative impact of procrastination compare to the negative academic impact of other issues that students face?

Beyond answering these questions, the present study also aims to shed light on other important aspects of student procrastination, including how it influences students’ distress, and how its prevalence and impact differ between undergraduate and graduate students.

## Methods

### Sample

The sample for this study contains 65,950 students from 97 US institutions of higher education, surveyed between January–May 2025 for the Spring 2025 American College Health Association-National College Health Assessment (ACHA-NCHA) [26–29]. This includes primarily 47,194 undergraduate students from 96 schools and 17,167 graduate & professional students from 81 schools, as well as 1,589 students who were in the full sample but in neither subgroup (e.g., because they were not pursuing a degree) [30–33].

Students in undergraduate degrees included those in all years of their degree (29.0% were in the 1^st^ year of undergrad, 23.3% in the 2^nd^, 25.4% in the 3^rd^, 18.9% in the 4^th^, and 3.5% in the 5^th^ year or more). Students in graduate and professional degrees included both those undertaking master’s degrees (57.6% of total, in degrees like MA, MS, MFA, and MBA) and doctoral degrees (42.4% of total, in degrees like PhD, EdD, MD, and JD). 91.5% of students were full-time (compared to 8.0% part-time, and 0.5% “Other”). The mean age of students was 23.2 years (median = 21.0), and 64.1% identified as cis women (27.5% as cis men, and 6.4% as transgender/non-conforming). Of the 97 institutions of higher education where students came from, 60 (61.9%) were public (compared to 38.1% private), and 92 (94.8%) were 4-year or above (compared to 5.2% 2-year).

Informed consent was obtained from participants during data collection for the NCHA, and participating schools also provided the necessary IRB and administrative approvals to the ACHA [34]. The data used for the analyses in the present study came from the aggregate reports publicly available at: https://www.acha.org/ncha/data-results/survey-results/academic-year-2024-2025/ accessed on 19-Sep-2025. The author had no access to information that could identify individual participants during or after data collection.

### Survey

As part of the ACHA-NCHA survey, students were asked whether they experienced problems or challenges with various types of issues in the last 12 months. The survey groups these issues into two broad categories. The first category contains 18 *life issues*, like procrastination, career, finances, appearance, family, peers, and bullying (or, in the case of ones like career, issues with that area of the student’s life). The second category contains 18 *health issues*, like anxiety, depression, ADHD, sleep difficulties, allergies, influenza, and injuries. For each of these issues that a student said they experienced, they were also asked about its impact on their academic performance, and, in the case of life issues, about its impact on their distress level too. For more details about the survey, including the specific questions students were asked as part of it, see the Supporting Information (SI).

### Analyses

The various life and health issues in the sample were assessed on three dimensions:

- *Prevalence*, representing the portion of students who reported suffering from the issue.
- *Performance*, representing the portion of students who reported that their academic performance was negatively impacted by the issue.
- *Distress*, representing the portion of students who reported significant distress because of the issue (available only for the life issues).

For both *performance* and *distress*, the relevant questions in the survey involved direct self-attribution of causation, as in “Within the last 12 months, to what extent did the following issue(s) cause you distress?”. For more information about this, see the SI.

The rates for these were calculated for three groups: undergraduate students, graduate & professional students, and all students. For performance and distress, two types of rates were calculated: one for the full sample of students and one for a conditional sample containing only students who indicated that they suffered from the issue in the prevalence question.

Procrastination’s prevalence, performance, and distress rates were then compared to those of the other life and health issues. This was done across the three student groups and for both full and conditional samples. Comparisons were conducted using a combination of significance tests (two-tailed chi-squared tests of proportion), bar charts (including 95% confidence intervals calculated using the Wilson method), and heatmaps.

No multiple-comparisons correction was applied, as the related analyses were descriptive and exploratory in nature, with a focus on the results of individual tests rather than a universal null hypothesis, and without placing emphasis on any result in particular in light of the size of its p-value [35,36]. At the same time, associated best-practice guidelines were followed for null hypothesis significance testing, including reporting exact p-values as well as effect sizes. From a practical perspective, however, this did not affect the study’s findings, since p-values for differences were small throughout (generally well below *p* < .001), mainly due to the large samples involved [37,38], which further drove a focus on descriptive analysis of effect sizes in the interpretation of the results.

Analyses were run using R [39] and RStudio [40]. The confidence intervals were calculated using the *binom* package [41], and the visualizations were constructed using *ggplot2* from the *tidyverse* [42], alongside *cowplot* [43] and *viridis* [44]. Packages were version-controlled for reproducibility using the *groundhog* package [45].

## Results

### Life issues

As Figs 1 and 2 show, out of all the life issues that students were asked about, procrastination was reported to be the most common, affecting 72.3% of all students (*ratio*_*prevalence*_ = 1.4 compared to the second most common issue, personal appearance, reported by 50.7% of students, *p* < .001). It was also the biggest cause of problems with academic performance, affecting 45.4% of all students (*ratio*_*performance*_ = 2.8 compared to the second biggest issue in this regard, finances, reported by 16.1% of students, *p* < .001), and the biggest cause of significant distress, affecting 45.7% of students (*ratio*_*distress*_ = 1.2 compared to the second biggest issue in this regard, academics, reported by 38.9% of students, *p* < .001).

**Fig 1:**
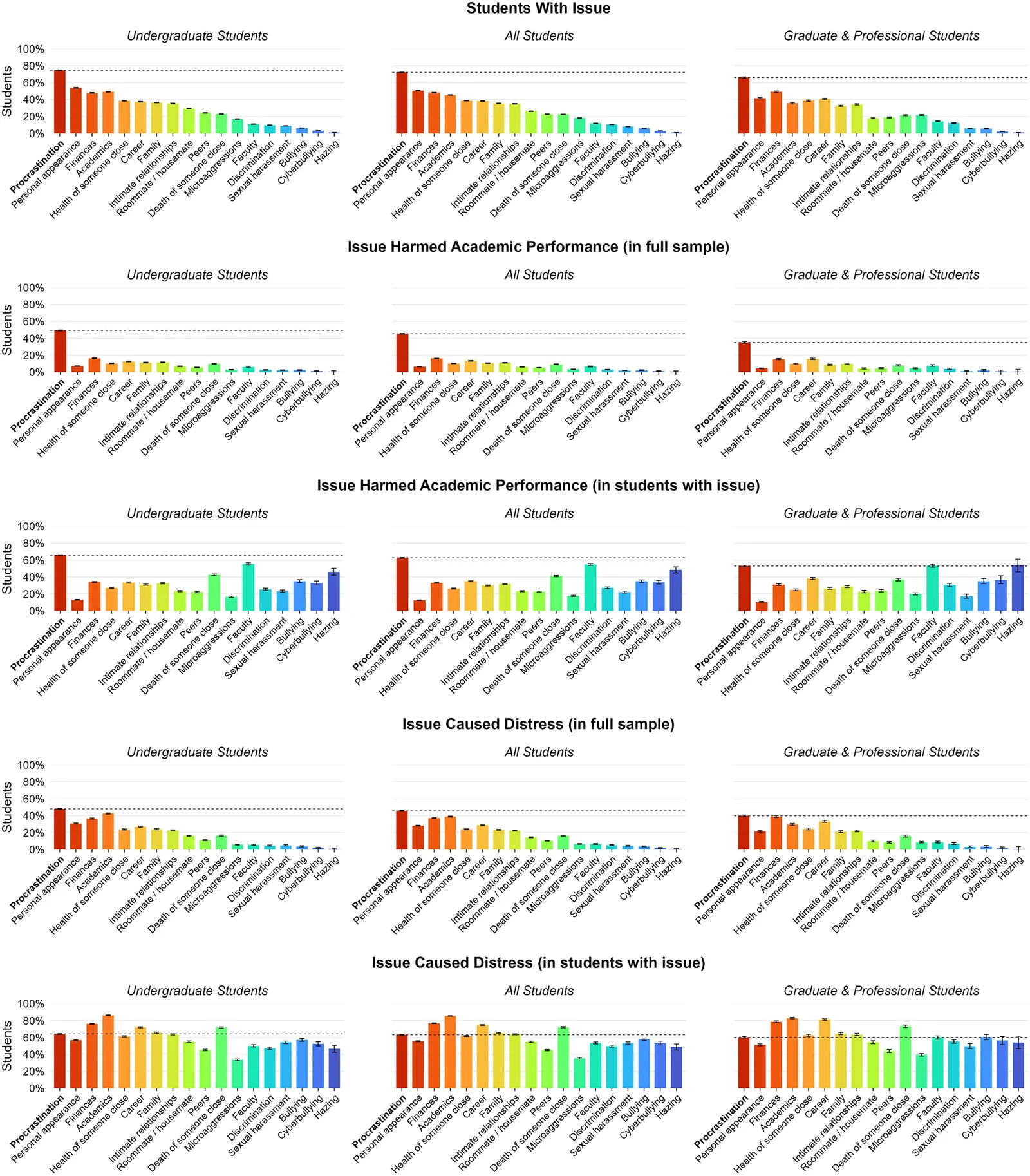
Bar charts showing the prevalence of life issues among students, the negative impact of those issues on students’ academic performance, and the distress caused by those issues. For impact and distress, percentages are shown both for full samples and conditional ones (among those who experienced that issue). Bars are sorted in decreasing order based on each issue’s prevalence among all students. Error bars are 95% confidence intervals. The dashed lines match the value of the procrastination bar in each chart.

**Fig 2:**
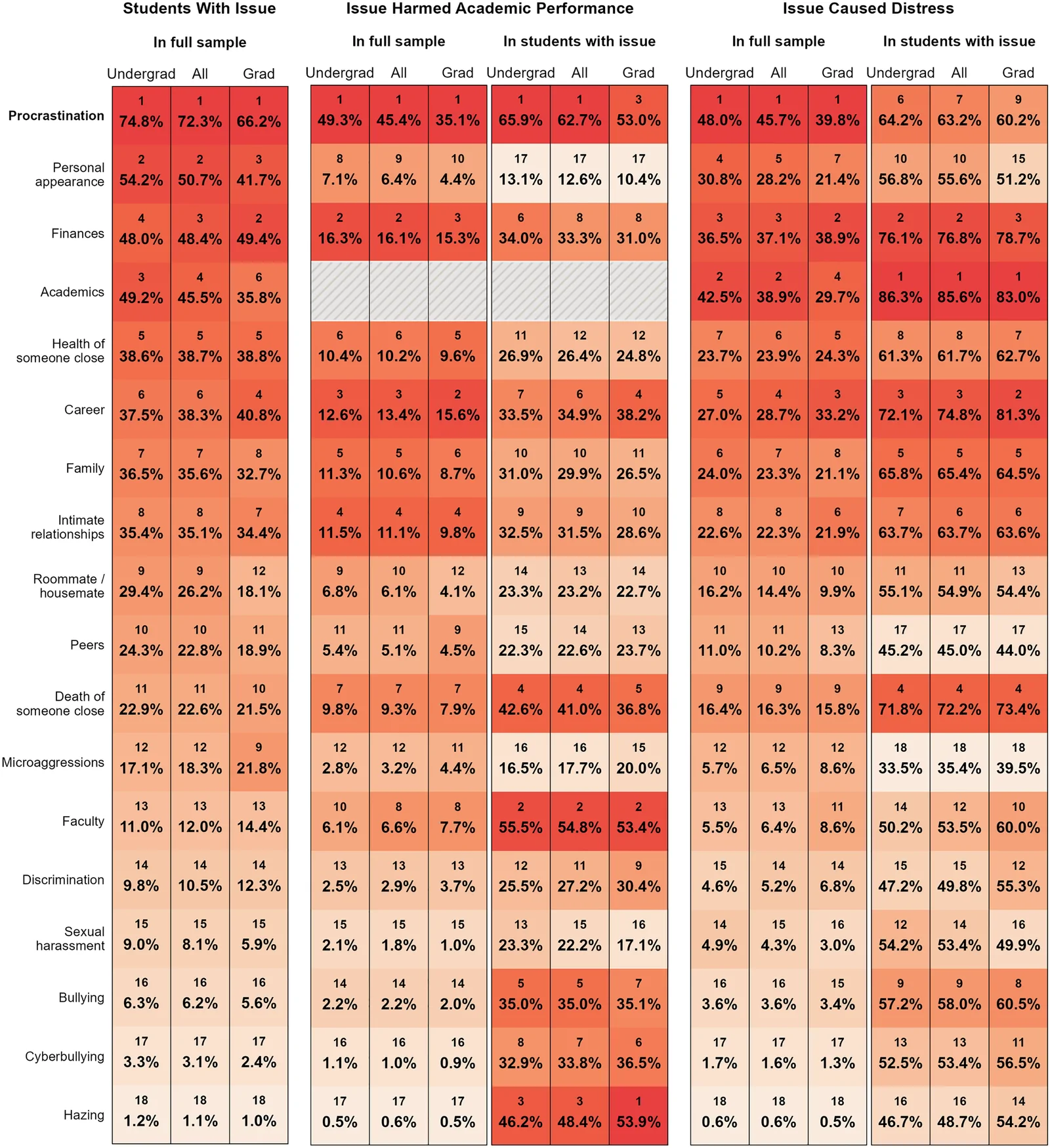
Heatmaps showing the prevalence of life issues among students, the negative impact of those issues on students’ academic performance, and the distress caused by those issues. For impact and distress, percentages are shown both for full samples and conditional ones (among those who experienced that issue). “Undergrad” refers to the sample with undergraduate students, “Grad” refers to the sample with graduate & professional students, and “All” refers to the combined sample with all students. Within each cell are the relevant percentage and the rank of that percentage within the column (darker red shading corresponds to a higher rank). Cells are sorted in decreasing order based on each issue’s prevalence among all students.

In the conditional samples, procrastination was still the leading cause of academic problems, negatively affecting the academic performance of 62.7% of students who reported experiencing it (*ratio*_*performance_conditional*_ = 1.1 compared to the second biggest issue in this regard, faculty, reported by 54.8% of students who had problems with it, *p* < .001). However, it was “only” the seventh biggest cause of distress conditionally, causing significant distress in 63.2% of students who suffered from it (*ratio*_*distress_conditional*_ = 0.7 compared to the biggest issue in this regard, academics, reported by 85.6% of students who had problems with it, *p* < .001).

In addition, as these Figs show, procrastination was reported to be a greater problem for undergraduate students than for graduate & professional students (*ratio*_*prevalence*_ = 1.1, from 74.8% versus 66.2%; *ratio*_*performance*_ = 1.4, from 49.3% versus 35.1%; *ratio*_*distress*_ = 1.2 from 48.0% versus 39.8%; all *p* < .001; *rank* of *p*rocrastination compared to other issues = 1 in all cases). Similarly, procrastination was a greater issue for undergraduates in the conditional samples, when it comes to its negative impacts on students who suffer from it (*ratio*_*performance_conditional*_ = 1.2, from 65.9% versus 53.0%, corresponding to *rank* = 1 versus 3; *ratio*_*distress_conditional*_ = 1.1, from 64.2% versus 60.2%, corresponding to *rank* = 6 versus 9; both *p* < .001).

### Health issues

As Figs 3 and 4 show, procrastination was more prevalent and detrimental to academic performance than a wide range of physical and mental health issues that students were asked about. Specifically, it was functionally equal in prevalence to the other top issue (affecting 72.3% of students compared to stress’s 73.8%, *ratio*_*prevalence*_ = 1.0, *p* < .001), was the top issue interfering with academic performance (harming 45.4% of students compared to stress as the second-biggest issue at 36.6%, *ratio*_*performance*_ = 1.2, *p* < .001), and was more prevalent and impactful than other top issues that students faced (all *p* < .001; see Fig 4 for percentages), like anxiety (*ratio*_*prevalence*_ = 1.1, *ratio*_*performance*_ = 1.5), depression (*ratio*_*prevalence*_ = 2.0, *ratio*_*performance*_ = 2.3), ADHD (*ratio*_*prevalence*_ = 3.6, *ratio*_*performance*_ = 3.5), and sleep difficulties (*ratio*_*prevalence*_ = 1.5, *ratio*_*performance*_ = 2.1). Note that here and elsewhere in the paper, the term “functionally equal” is used when the ratio between the prevalence or impact of procrastination and another issue was close enough to a null value to be reported as 1.0 (as in the case of the prevalence of procrastination and stress, where the *ratio*_*prevalence*_ was 0.723/0.738 ≈ 1.0). This focus on effect size when there is high similarity between the two values is particularly important given this study’s large sample, since “overpowered” tests often indicate even tiny differences as statistically significant [37,38].

**Fig 3:**
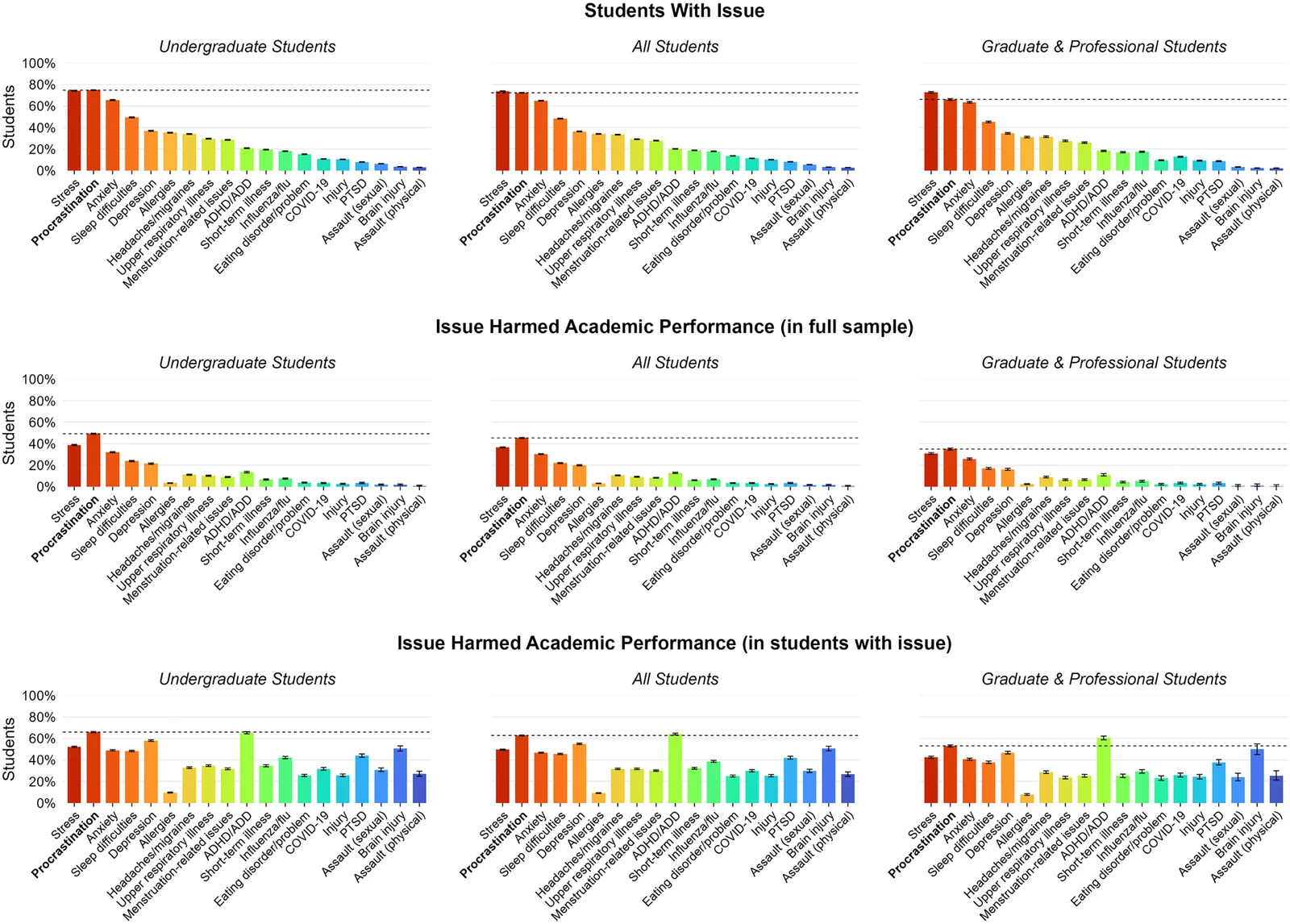
Bar charts showing the prevalence of health issues among students, and the negative impact of those issues on students’ academic performance. For impact, percentages are shown both for full samples and conditional ones (among those who experienced that issue). Bars are sorted in decreasing order based on each issue’s prevalence among all students. Error bars are 95% confidence intervals. The dashed lines match the value of the procrastination bar in each chart.

**Fig 4:**
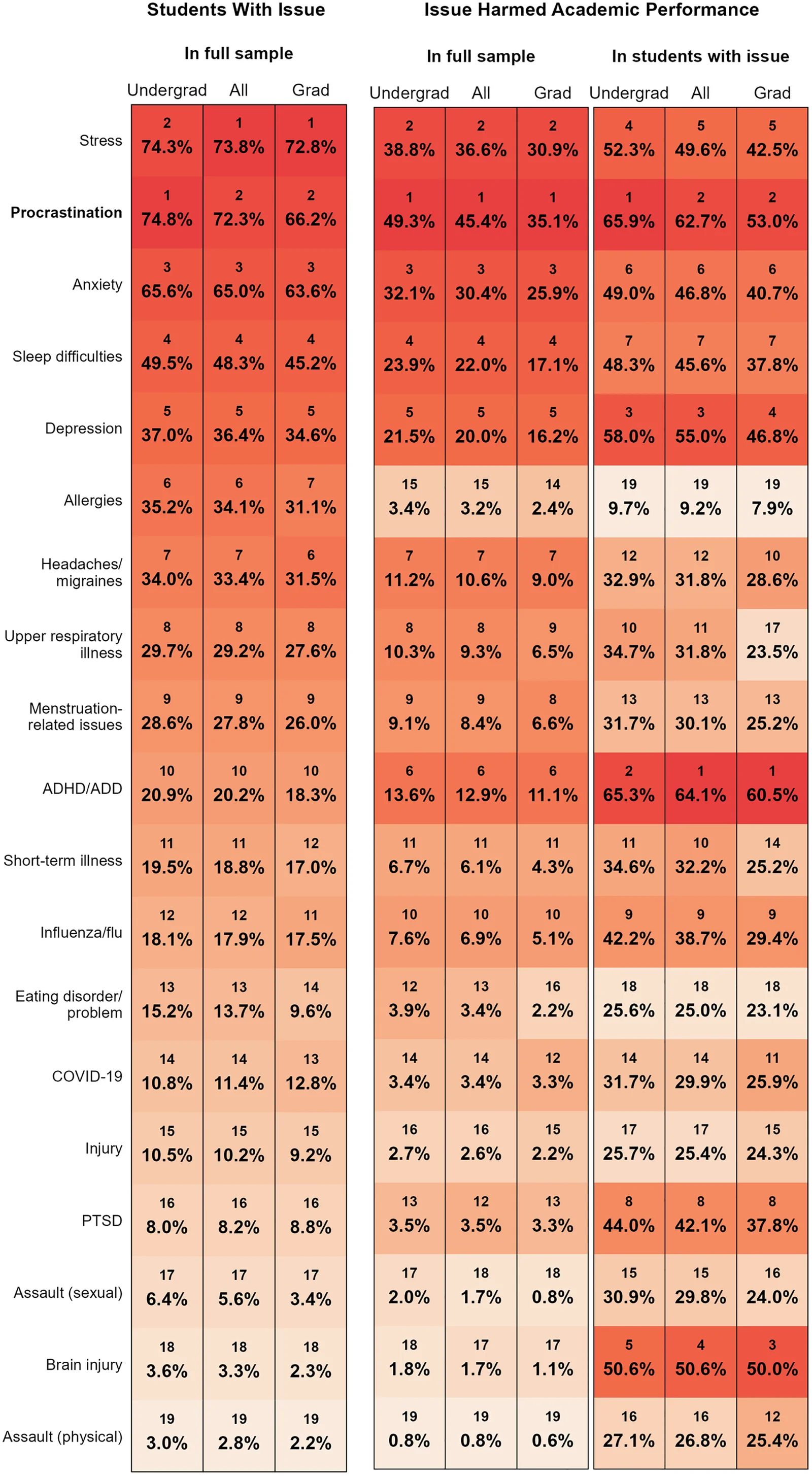
Heatmaps showing the prevalence of health issues among students, and the negative impact of those issues on students’ academic performance. For impact, percentages are shown both for full samples and conditional ones (among those who experienced that issue). “Undergrad” refers to the sample with undergraduate students, “Grad” refers to the sample with graduate & professional students, and “All” refers to the combined sample with all students. Within each cell are the relevant percentage and the rank of that percentage within the column (darker red shading corresponds to a higher rank). Cells are sorted in decreasing order based on each issue’s prevalence among all students.

In conditional terms, procrastination was functionally equal to the top issue (affecting 62.7% of students who experienced it compared to 64.1% for ADHD, *ratio*_*performance_conditional*_ = 1.0, *p* = .004). Finally, as with the life issues, procrastination was a greater issue for undergraduates than for graduate & professional students in terms of rank compared to other issues, but it was still a leading issue for both groups (*rank*_*prevalence*_ = 1 versus 2, *rank*_*performance*_ = 1 for both, and *rank*_*performance_conditional*_ = 1 versus 2).

## Discussion

This study investigated the prevalence and impact of procrastination, and compared them to those of a wide range of life issues (around areas like career, finances, appearance, family, and bullying) and health issues (like anxiety, depression, ADHD, sleep difficulties, and influenza), among over 65,000 students enrolled in higher education.

Procrastination was found to be the most common issue that students reported (functionally tied with stress). Specifically, it caused problems to 72.3% of all students in the last 12 months (and 74.8% of undergraduates), which roughly replicated past findings on this, such as the finding that around 75% of undergraduate students consider themselves procrastinators. Procrastination was also the issue that most often harmed students’ academic performance, affecting 45.4% of them, which was several times as often as issues like finances (2.8x) and ADHD (3.5x). And, procrastination was also the most common cause of significant distress compared to the other life issues (distress was not measured for health issues), affecting 45.7% of students. The prominence of these negative effects applied both to undergraduate students and to graduate & professional ones, although undergraduates consistently suffered more from procrastination. This could be attributable to various causes, such as: age differences, with younger people procrastinating more on average [3,7]; selection effects, in terms of which students proceed to advanced degrees; and structural differences across educational programs, when it comes to factors like the level of autonomy given to students, the degree of external accountability students have, the expected standards for students’ performance, and the types of academic tasks and deadlines that students are assigned.

There are several important caveats to these findings.

First, although procrastination had the biggest impact in the full samples, other issues sometimes had bigger impact in conditional terms (among students who suffered from any given issue), especially when it comes to graduate & professional students and to distress. The reason for this apparent discrepancy is that even in cases where procrastination did not have the highest conditional impact, its conditional impact was still high enough that, when combined with its high prevalence, its total impact ranked at or near the top of all issues, indicating a high relative burden within each sample. This highlights how differences in the total impact of issues across populations—in this case of procrastination among undergraduate students compared to graduate & professional ones—are attributable to differences in both the prevalence of each issue and its conditional impact. This is also an important reminder that even if a certain issue only affects a small subset of students, its impact can be significant for those students. A key example of this is ADHD: despite having similar conditional impact on students’ academic performance as procrastination (64.1% compared to 62.7%), it affected the academic performance of “only” 12.9% of students compared to procrastination’s 45.4%, because only 20.2% of students experienced it compared to procrastination’s 72.3% (as shown in Fig 4).

Second, there is uncertainty regarding the impact of procrastination and the other issues. For one, their impact was measured in terms of the portion of students affected, rather than the extent to which each student was affected, although a follow-up analysis (presented in the SI) replicated the findings regarding the impact of life issues on distress using a 0–3 scale. For another, there are various potential relationships between the different issues, which could influence the impact of any given issue. Stress, for instance, was a top issue, but procrastination was a top cause of distress, and procrastination itself can be caused or exacerbated by this and other issues, like anxiety and lack of sleep, potentially—but not necessarily—in a bidirectional manner that results in vicious cycles [1,46–49]. An example of such a feedback loop is a student procrastinating on a task, which causes them to feel stressed, which interferes with their sleep, which causes them to procrastinate on the task further.

These potential interplays add an element of uncertainty when comparing the impact of the different issues that students face, since it is unclear to what extent students’ reporting accounts for such higher-order effects. Notably, it is possible that any given issue has greater impact on students than it initially appears to, because it is the upstream cause of downstream issues that are then reported to hurt students’ academic performance or cause them distress. For example, it is possible that a portion of the negative effect of students’ stress on their academic performance should be attributed to procrastination, and that, at the same time, a portion of procrastination’s own negative effect should be partially attributed to ADHD.

From a practical perspective, the key concern here is that we might under- or over-estimate the impact of different issues, and mainly that we will prioritize issues that are symptoms of other issues, in cases where treating the underlying cause is more important. To address these uncertainties, it would help to conduct further research into when and to what extent these factors cause and are caused by procrastination, and to what degree these higher-order effects are reflected in students’ reporting. Nevertheless, although these uncertainties necessitate added caution when interpreting the impact of the issues that were examined in this study, the present findings represent at least first-order approximations of impact, which are a useful starting point for current prioritization and for any future work.

Finally, the survey used in the study had additional limitations. In terms of measurements, it relies on a single-item measure of procrastination, as opposed to the validated scales of procrastination used in most past literature, such as the *Pure Procrastination Scale* and the *Tuckman Procrastination Scale* [24,50]. In addition, although the survey involved a sample that is large and diverse in various senses, some populations are under-/over-represented in it [51,52]. Furthermore, its self-reporting nature could cause issues, such as students underestimating the extent to which procrastination affects them, or the worst procrastinators not getting around to filling out the survey. As Lederer et al. [53, p. 1137] note: “Although the ACHA-NCHA III is the best available data source for comprehensive college health information, it is not representative of all U.S. college students or institutions of higher education.” And, its results might not directly apply to other types of students, such as those outside the US.

Future research could potentially address these limitations in various ways, for example by assessing these issues in other types of samples. Future research could also investigate related topics of interest, such as the cost of addressing these issues, which is important for deciding how to prioritize academic interventions given limited resources.

Nevertheless, despite its limitations, this study provides novel insights into the prevalence and impact of procrastination, while contextualizing them against the prevalence and impact of many other issues that students face. Its findings suggest that it may be insufficient to simply think of procrastination as *a* big issue among students; rather, it seems plausible that procrastination is *the* biggest issue on average, at least in terms of its impact on students’ academic performance. This aligns with research on procrastination in the workplace, which found a roughly $20,000 drop in average salary for every 1-point increase on a 5-point procrastination scale, leading the researchers to conclude that “procrastination could be the most important personality trait associated with income that we have yet identified, perhaps second to agreeableness” [54, p. 395] (in 2025 dollars, adjusted from 2013). This, in turn, suggests that the harmful effects of procrastination persist after students finish their schooling, whether because students’ academic struggles interfered with their ability to get certain roles in a competitive job market, because procrastination continues to interfere with graduates’ ability to advance in the workplace, or because of some other combination of mechanisms.

Fortunately, various interventions are effective in reducing procrastination [55–57], and there are many practical techniques, like breaking down complex assignments and encouraging reflection, that educators can use to help their students take action on time [9]. Implementing these techniques and interventions—and teaching students to self-regulate their own behavior in the long-term—is crucial for students’ wellbeing and success, both in school and after they graduate.

## Supporting information

S1 File Supporting Information. (PDF)
