# Supplementary material for: Procrastination among US college students: Prevalence and impact relative to common health and life issues
Source: PLoS One. 2026 Oct 7;21(10):e0357714. doi: 10.1371/journal.pone.0357714 (PMC13645087; doi:10.1371/journal.pone.0357714)
Supplement: Supplementary file 1 [file pone.0357714.s001.pdf]

Supporting Information for:

Procrastination among US college students: Prevalence and impact relative to common health and life issues

Additional methodological information

Survey questions

For life issues, students were asked the following questions:

- *Prevalence*- "Within the last 12 months, have you had problems or challenges with any of the following?"
- *Performance*- "Within the last 12 months, to what extent did the following issue(s) negatively affect your academic performance?" This was asked using conditional logic about issues that each student said they faced in the Prevalence questions. The options for responses were "Did not affect academic performance", "Negatively impacted performance in a class", and "Delayed progress towards degree". The ACHA primarily analyzes these in a binary way to determine whether any given problem negatively impacted a student's academic performance, where the first response is treated as "No", while the latter two responses are combined and treated as "Yes".
- *Distress*- "Within the last 12 months, to what extent did the following issue(s) cause you distress?" This too was asked about issues that students said they faced in the Prevalence question. The four possible responses were No Distress, Minimal Distress, Moderate Distress, and High Distress. The ACHA primarily analyzes these in a binary way too, differentiating between No/Minimal Distress and Moderate/High Distress.

For health issues, there was no distress question, and both prevalence and performance were based on responses to the question "Within the last 12 months, have any of the following affected your academic performance?" where one of the available responses were "Did not experience this issue". Where applicable, issues in this category were asked about in a mutually exclusive way; for example, the question for the *injury* category is meant to exclude cases covered under *brain injury*.

Survey responses

Informed consent was obtained from participants during data collection for the NCHA, and participating schools also provided the necessary IRB and administrative approvals to the ACHA [1]. The data used for the present analyses came from the aggregate reports publicly available at: https://www.acha.org/ncha/data-results/survey-results/academic-year-2024-2025/

The rates used in the present analyses were calculated using the number of students who had a valid response for any given question, and validity rates were very high across all questions and student groups (*mean* = 99.0%, *range* = 97.1%–99.8%).[1] By contrast, the mean response rate among students in the schools was reported by the ACHA to be 11% (*median* = 10%). This is an important caveat, as it could mean, for instance, that the worst procrastinators tended to not fill out the survey in the first place. While a low response rate does not inherently cause issues for analyses [2–4], it does tie in to other potential limitations of the ACHA-NCHA, which, despite being uniquely large and diverse in many senses, is not fully representative of the underlying student population in terms of factors like demographics [5–7]. While it is not clear that this has practical implications on various analyses of the data in the NCHA [8,9], especially in light of ongoing improvements that were made to the survey [10], it does necessitate caution when interpreting its findings.

Calculation of impact rates

In the case of health issues, impact rates (on academic performance) were calculated by taking the number of people within a sample who said that an issue negatively affected them and dividing it by the number of people who had a valid response for the question about that issue. This means, for example, that the rate of impact of anxiety on academic performance among all students was:

$$19{,}479 \div 64{,}109 \approx 30.4\%$$

In the case of life issues, impact rates (on academic performance and distress) were calculated by taking the prevalence rate for any given combination of issue and sample and

[1] The one exception was the final question about health issues, which asked whether participants experienced other issues not captured by previous questions. This question had a 67.2% validity rate, so it was excluded from the analyses. Out of those who did respond to this question, only 2.4% of those who responded said that they experienced other issues, making this the least commonly prevalent issue. By contrast, this category had high conditional impact on academic performance (62.8%), though it affected the academic performance of only 1.5% of students due to its low prevalence.

multiplying it by the corresponding conditional rate for that combination (which itself was based on the proportion of students who said that an issue affected them negatively out of all students who had a valid response for the relevant question about that issue and negative effect). This means, for example, that the rate of impact of procrastination on academic performance among all students was roughly:

$$72.32\% \times 62.72\% \approx 45.4\%$$

The reason these rates were calculated in slightly different ways for life and health issues is that, as noted above, students were asked about the two types of issues in slightly different ways. Specifically, in the case of health issues, both the prevalence and impact of each issue were assessed in a single question. Conversely, in the case of life issues, there were separate questions for prevalence and impact, and the number of people who had a valid response for each impact question was slightly lower than for the corresponding prevalence question. For example, if the rate of impact of procrastination on academic performance was calculated by dividing the number of people who said that procrastination negatively affected their academic performance by the number of people who had a valid response to the prevalence question for procrastination, the result would be:

$$29{,}327 \div 64{,}934 \approx 45.2\%$$

The difference between this and the previous rate stems from the fact that of the 46,962 students who said that they experienced procrastination in the prevalence question, 46,761 had a valid response to the corresponding question about the impact of procrastination on their academic performance (a difference of 201), out of which 62.7% indicated that procrastination negatively impacted their academic performance. As this shows, however, the results are functionally identical regardless of the calculation method (45.4% compared to 45.2% in the case of procrastination), due to the consistently high validity rates across questions.

In addition, note that the ACHA's executive summaries use a denominator-based approach with the number of all students in a sample as the denominator, regardless of the number of valid responses for each question. However, given the consistently high rate of valid responses, this too makes functionally no difference to the present analyses. For example, the rate at which procrastination was reported to harm students' academic performance among all students is 45.4% here and 44.5% in the corresponding executive summary (due to the use of 65,950 as the denominator), while the rate of the second most

academically harmful life issue (finances) is 16.1% here and 15.8% in the executive summary, and the rate of the second most harmful health issue (stress) is 36.6% here and 35.8% in the executive summary [11].

Renaming of issues

Some of the names of the issues were modified from the original in the figure labels for presentational purposes, as indicated in Table S1. The ACHA uses similar presentational modifications in their executive summaries, as in the case of ‘Post Traumatic Stress Disorder (PTSD)’ → ‘PTSD’ [11].

Table S1. Issue names that were modified in this study's figures for presentational purposes.

Issue type	Original phrasing	Modified phrasing
Life	Death of a family member, friend, or someone close to me	Death of someone close
Life	Health of someone close to me	Health of someone close
Life	I experienced discrimination	Discrimination
Life	I experienced microaggression(s)	Microaggressions
Life	I was bullied	Bullying
Life	I was cyberbullied	Cyberbullying
Life	I was hazed	Hazing
Life	I was sexually harassed	Sexual harassment
Health	Attention-Deficit/Hyperactivity Disorder (ADHD) or Attention-Deficit Disorder (ADD)	ADHD/ADD
Health	Concussion or Traumatic Brain Injury (TBI)	Brain injury
Health	Influenza or influenza like illness (the flu)	Influenza/flu
Health	Injury (for example: burn, sprain, or broken bone) excluding concussion or TBI	Injury
Health	PMS (Premenstrual Syndrome), painful periods, or menstrual cramping	Menstruation-related issues
Health	Post Traumatic Stress Disorder (PTSD)	PTSD
Health	Short-term illness, excluding upper respiratory illness and influenza	Short-term illness
Health	Upper respiratory illness (for example: sinus infection, colds, or sore throat, etc.)	Upper respiratory illness

Analysis of mean distress

A follow-up analysis was conducted to check the impact of the life issues on distress using a 0–3 scale (where No Distress = 0, Minimal Distress = 1, Moderate Distress = 2, and High Distress = 3), instead of the binary scale used in the main analysis (where No/Minimal Distress are contrasted with Moderate/High Distress).

Figure S1 contains the heatmaps for this follow-up analysis. It shows that the results using this scale were highly similar to those using the binary scale. This was confirmed through additional correlations (for the All Students group), since *Pearson's r* for the association between mean distress caused by an issue and the portion of students who experienced significant distress from that issue was ~0.99 in both the full and conditional samples, as was the *Spearman's rho* for the corresponding ranks of the various issues.

This Figure also shows that procrastination was the number 1 cause of distress across all groups when using this scale too. And, the impact of procrastination compared to the second-biggest cause of distress (academics) was slightly greater here than when using the binary scale ($ratio_{distress}$ = 1.3 versus 1.2). In the conditional samples, procrastination was ranked 1 spot lower for the Undergraduate and Graduate groups, although its conditional impact compared to the biggest conditional cause of distress (academics) was slightly greater here than when using the binary scale ($ratio_{distress\_conditional}$ = 0.8 versus 0.7).

Figure S1. Heatmaps showing the prevalence of life issues among students, the negative impact of those issues on students' academic performance, and the distress caused by those issues. For impact and distress, percentages are shown both for full samples and conditional ones (among those who experienced that issue). "Undergrad" refers to the sample with undergraduate students, "Grad" refers to the sample with graduate & professional students, and "All" refers to the combined sample with all students. Within each cell are the relevant percentage and the rank of that percentage within the column (darker red shading corresponds to a higher rank). Cells are sorted in decreasing order based on each issue's prevalence among all students.

	Students With Issue			Issue Harmed Academic Performance						Mean Distress Caused by Issue					
	In full sample			In full sample			In students with issue			In full sample			In students with issue		
	Undergrad	All	Grad	Undergrad	All	Grad	Undergrad	All	Grad	Undergrad	All	Grad	Undergrad	All	Grad
**Procrastination**	1<br>74.8%	1<br>72.3%	1<br>66.2%	1<br>49.3%	1<br>45.4%	1<br>35.1%	1<br>65.9%	1<br>62.7%	3<br>53.0%	1<br>1.39	1<br>1.33	1<br>1.18	7<br>1.86	7<br>1.84	10<br>1.78
Personal appearance	2<br>54.2%	2<br>50.7%	3<br>41.7%	8<br>7.1%	9<br>6.4%	10<br>4.4%	17<br>13.1%	17<br>12.6%	17<br>10.4%	4<br>0.94	4<br>0.87	6<br>0.68	9<br>1.74	11<br>1.72	14<br>1.63
Finances	4<br>48.0%	3<br>48.4%	2<br>49.4%	2<br>16.3%	2<br>16.1%	3<br>15.3%	6<br>34.0%	8<br>33.3%	8<br>31.0%	3<br>1.01	3<br>1.02	2<br>1.05	2<br>2.09	2<br>2.10	3<br>2.12
Academics	3<br>49.2%	4<br>45.5%	6<br>35.8%							2<br>1.12	2<br>1.03	4<br>0.79	1<br>2.27	1<br>2.26	1<br>2.21
Health of someone close	5<br>38.6%	5<br>38.7%	5<br>38.8%	6<br>10.4%	6<br>10.2%	5<br>9.6%	11<br>26.9%	12<br>26.4%	12<br>24.8%	7<br>0.69	6<br>0.70	5<br>0.71	8<br>1.80	8<br>1.81	7<br>1.82
Career	6<br>37.5%	6<br>38.3%	4<br>40.8%	3<br>12.6%	3<br>13.4%	2<br>15.6%	7<br>33.5%	6<br>34.9%	4<br>38.2%	5<br>0.75	5<br>0.79	3<br>0.89	4<br>1.99	4<br>2.05	2<br>2.19
Family	7<br>36.5%	7<br>35.6%	8<br>32.7%	5<br>11.3%	5<br>10.6%	6<br>8.7%	10<br>31.0%	10<br>29.9%	11<br>26.5%	6<br>0.70	7<br>0.67	8<br>0.61	5<br>1.91	5<br>1.89	6<br>1.86
Intimate relationships	8<br>35.4%	8<br>35.1%	7<br>34.4%	4<br>11.5%	4<br>11.1%	4<br>9.8%	9<br>32.5%	9<br>31.5%	10<br>28.6%	8<br>0.66	8<br>0.66	7<br>0.64	6<br>1.88	6<br>1.87	5<br>1.87
Roommate / housemate	9<br>29.4%	9<br>26.2%	12<br>18.1%	9<br>6.8%	10<br>6.1%	12<br>4.1%	14<br>23.3%	13<br>23.2%	14<br>22.7%	9<br>0.51	10<br>0.45	10<br>0.31	10<br>1.73	10<br>1.73	11<br>1.71
Peers	10<br>24.3%	10<br>22.8%	11<br>18.9%	11<br>5.4%	11<br>5.1%	9<br>4.5%	15<br>22.3%	14<br>22.6%	13<br>23.7%	11<br>0.37	11<br>0.34	12<br>0.28	16<br>1.52	16<br>1.51	17<br>1.49
Death of someone close	11<br>22.9%	11<br>22.6%	10<br>21.5%	7<br>9.8%	7<br>9.3%	7<br>7.9%	4<br>42.6%	4<br>41.0%	5<br>36.8%	10<br>0.47	9<br>0.46	9<br>0.45	3<br>2.06	3<br>2.06	4<br>2.07
Microaggressions	12<br>17.1%	12<br>18.3%	9<br>21.8%	12<br>2.8%	12<br>3.2%	11<br>4.4%	16<br>16.5%	16<br>17.7%	15<br>20.0%	12<br>0.22	12<br>0.24	11<br>0.31	18<br>1.29	18<br>1.32	18<br>1.41
Faculty	13<br>11.0%	13<br>12.0%	13<br>14.4%	10<br>6.1%	8<br>6.6%	8<br>7.7%	2<br>55.5%	2<br>54.8%	2<br>53.4%	13<br>0.18	13<br>0.20	13<br>0.26	14<br>1.61	12<br>1.67	8<br>1.81
Discrimination	14<br>9.8%	14<br>10.5%	14<br>12.3%	13<br>2.5%	13<br>2.9%	13<br>3.7%	12<br>25.5%	11<br>27.2%	9<br>30.4%	15<br>0.15	14<br>0.17	14<br>0.21	15<br>1.53	15<br>1.58	12<br>1.69
Sexual harassment	15<br>9.0%	15<br>8.1%	15<br>5.9%	15<br>2.1%	15<br>1.8%	15<br>1.0%	13<br>23.3%	15<br>22.2%	16<br>17.1%	14<br>0.15	15<br>0.14	16<br>0.09	12<br>1.68	13<br>1.67	16<br>1.59
Bullying	16<br>6.3%	16<br>6.2%	16<br>5.6%	14<br>2.2%	14<br>2.2%	14<br>2.0%	5<br>35.0%	5<br>35.0%	7<br>35.1%	16<br>0.11	16<br>0.11	15<br>0.10	11<br>1.73	9<br>1.74	9<br>1.78
Cyberbullying	17<br>3.3%	17<br>3.1%	17<br>2.4%	16<br>1.1%	16<br>1.0%	16<br>0.9%	8<br>32.9%	7<br>33.8%	6<br>36.5%	17<br>0.05	17<br>0.05	17<br>0.04	13<br>1.64	14<br>1.65	13<br>1.69
Hazing	18<br>1.2%	18<br>1.1%	18<br>1.0%	17<br>0.5%	17<br>0.6%	17<br>0.5%	3<br>46.2%	3<br>48.4%	1<br>53.9%	18<br>0.02	18<br>0.02	18<br>0.02	17<br>1.42	17<br>1.47	15<br>1.62

References

1. American College Health Association. ACHA-NCHA web version frequently asked questions [Internet]. 2024. Available from: https://portal.acha.org/documents/ncha/ACHA-NCHA-WEB_FAQ_FINAL_FEB_2024.pdf

2. Holtom B, Baruch Y, Aguinis H, A Ballinger G. Survey response rates: Trends and a validity assessment framework. Hum Relations. 2022;75(8):1560–84.

3. Massey DS, Tourangeau R. Where do we go from here? Nonresponse and social measurement. Ann Am Acad Pol Soc Sci. 2013;645(1):222–36.

4. Fosnacht K, Sarraf S, Howe E, Peck LK. How important are high response rates for college surveys? Rev High Educ. 2017;40(2):245–65.

5. Giovenco DP, Gundersen DA, Delnevo CD. Reaching a representative sample of college students: A comparative analysis. J Am Coll Heal. 2016;64(3):262–7.

6. Jackson ZA, Sherman LD. National college health assessment sampling: Organizational barriers preventing black participation and organizational solutions for appropriate representation. J Am Coll Heal. 2019;67(6):501–4.

7. Rahn RN, Pruitt B, Goodson P. Utilization and limitations of the American College Health Association’s National College Health Assessment instrument: A systematic review. J Am Coll Heal. 2016;64(3):214–37.

8. Kerr DCR, Bae H, Alley ZM. Enhancing gender and ethnic representativeness of NCHA-II data with survey weights: The examples of substance use prevalence and state marijuana legalization. J Am Coll Heal [Internet]. 2021;69(4):370–7. Available from: https://doi.org/10.1080/07448481.2019.1679151

9. American College Health Association. ACHA-NCHA-II reliability and validity analyses. Hanover, MD: American College Health Association; 2013.

10. Lederer AM, Hoban MT. The development of the American College Health Association-National College Health Assessment III: An improved tool to assess and enhance the health and well-being of college students. J Am Coll Heal. 2022;70(6):1606–10.

11. American College Health Association. American College Health Association-National

College Health Assessment III: Reference Group Executive Summary Spring 2025 [Internet]. Silver Spring, MD; 2025. Available from: https://www.acha.org/wp-content/uploads/NCHA-IIIb_SPRING_2025_REFERENCE_GROUP_INSTITUTIONAL_EXECUTIVE_SUMMARY.pdf
