# Supplementary material for: Procrastination among US college students: Prevalence and impact relative to common health and life issues
Source: PLoS One. 2026 Oct 7;21(10):e0357714. doi: 10.1371/journal.pone.0357714 (PMC13645087; doi:10.1371/journal.pone.0357714)
Supplement: Supplementary file 2 [file pone.0357714.s003.docx]

Dear editor and reviewers,

Thank you for the thoughtful comments and for the opportunity to revise the manuscript! I appreciate them both.

Below, I explain how I revised the manuscript to address your comments. For concision and clarity, I included only comments with suggestions for the manuscript (e.g., as opposed to ones summarizing it or discussing its strengths).

# **REVIEWER 1**

*> The manuscript states that procrastination and stress are "functionally equal in prevalence" despite a statistically significant difference (72.3% vs. 73.8%, p < .001). Given the large sample size, statistical significance may not necessarily indicate practical significance. The authors should either justify the use of the term "functionally equal" by discussing practical equivalence or rephrase the statement as "similar in prevalence" and consider reporting an effect size.*

— I added the following clarification: “Note that here and elsewhere in the paper, the term “functionally equal” is used when the ratio between the prevalence or impact of procrastination and another issue was close enough to a null value to be reported as 1.0 (as in the case of the prevalence of procrastination and stress, where the *ratio_prevalence_* was 0.723/0.738 ≈ 1.0). This focus on effect size when there is high similarity between the two values is particularly important given this study’s large sample, since “overpowered” tests often indicate even tiny differences as statistically significant [37,38].”

In addition, the impact of large samples on p-values and the implications of this for the analyses are now mentioned explicitly in the Methods (see the response to comment #2 by reviewer 2). All these edits also aim to take into account the editor’s related comment (“*For instance, consider the issue of potentially overpowered tests leading to conclusions that, while significant, may be practically useless*.”) and the relevant assessment by reviewer 2 in their original review (“*The authors appropriately recognize that with N = 65,950, p-values alone are uninformative, and correctly pivot to ratios and rank comparisons as the substantively meaningful metrics (e.g., explicitly calling stress and procrastination "functionally equal" in prevalence despite p < .001). This shows good statistical judgment.*”).

*> One of the most interesting findings is the discrepancy between procrastination's ranking in overall distress and its lower ranking in conditional distress. Expanding the discussion of why procrastination imposes such a large population-level burden despite not producing the highest conditional distress would strengthen the practical implications for intervention prioritization.*

— I have expanded the discussion on this as suggested. The relevant part now reads:

“First, although procrastination had the biggest impact in the full sample (among all students), other issues sometimes had bigger impact in conditional terms (among students who suffered from any given issue), especially when it comes to graduate & professional students and to distress. The reason for this apparent discrepancy is that even in cases where procrastination did not have the highest conditional impact, its conditional impact was still high enough that, when combined with its prevalence, its total impact ranked at or near the top of all issues, indicating a high relative burden within each population. This highlights how differences in the total impact of issues across populations—in this case of procrastination among undergraduate students compared to graduate & professional ones—are attributable to differences in both the prevalence of each issue and its conditional impact. This is also an important reminder that even if a certain issue only affects a small subset of students, its impact can be significant for those students. A key example of this is ADHD: despite having similar conditional impact on students’ academic performance as procrastination (64.1% compared to 62.7%), it affected “only” 12.9% of students compared to procrastination’s 45.4%, because only 20.2% of students experienced it compared to procrastination’s 72.3% (as shown in Fig 4).”

*> Although the manuscript acknowledges potential bidirectional relationships between procrastination and factors such as stress, anxiety, and sleep difficulties, a more explicit discussion of how these overlapping and reciprocal relationships may influence interpretation of the comparative findings would add theoretical depth.*

— Added an explicit discussion on this:

“For another, there are various potential relationships between the different issues, which could influence the impact of any given issue. Stress, for instance, was a top issue, but procrastination was a top cause of distress, and procrastination itself can be caused or exacerbated by this and other issues, like anxiety and lack of sleep, potentially—but not necessarily—in a bidirectional manner that results in vicious cycles [1,46–49]. An example of such a feedback loop is a student procrastinating on a task, which causes them to feel stressed, which interferes with their sleep, which causes them to procrastinate on the task further.

These potential interplays add an element of uncertainty when comparing the impact of the different issues that students face, since it is unclear to what extent students’ reporting accounts for such higher-order effects. Notably, it is possible that any given issue has greater impact on students than it initially appears to, because it is the upstream cause of downstream issues that are then reported to hurt students’ academic performance or cause them distress. For example, it is possible that a portion of the negative effect of students’ stress on their academic performance should be attributed to procrastination, and that, at the same time, a portion of procrastination’s own negative effect should be partially attributed to ADHD.

From a practical perspective, the key concern is that we might under- or over-estimate the impact of different issues, and mainly that we will prioritize issues that are symptoms of other issues, in cases where treating the underlying cause is more important. To address these uncertainties, it would help to conduct further research into when and to what extent these factors cause and are caused by procrastination, and to what degree these higher-order effects are reflected in students’ reporting. Nevertheless, although these uncertainties necessitate added caution when interpreting the impact of the issues that were examined in this study, the present findings represent at least first-order approximations of impact, which are a useful starting point for current prioritization and for any future work.”

*> The discussion suggests that lower rates of procrastination among graduate and professional students may reflect age-related maturity or selection effects. It may also be worthwhile to consider whether structural differences in graduate education, such as greater autonomy and fewer short-term assignment deadlines, contribute to these observed differences.*

— I updated the discussion to reflect that these and additional structural factors could play a role in the difference in procrastination rates across the two student groups. The relevant part now reads: “The prominence of these negative effects applied both to undergraduate students and to graduate & professional ones, although undergraduates consistently suffered more from procrastination. This could be attributable to various causes, such as: age differences, with younger people procrastinating more on average [3,7]; selection effects, in terms of which students proceed to advanced degrees; and structural differences across educational programs, when it comes to factors like the level of autonomy given to students, the degree of external accountability students have, the expected standards for students’ performance, and the types of academic tasks and deadlines that students are assigned.”

*> Figures are currently placed after the reference section. While this is acceptable for review purposes, they should be appropriately integrated or clearly indicated in the final publication format.*
­­— The location of the figures is now clearly indicated in the body via their captions per PLOS’s requirements, with the final placement and integration of the figure files into the manuscript confirmed to be handled by PLOS’s production team before publication.

# REVIEWER 2

*> 1. Clarify whether harm/distress items involve direct self-attribution of causation, to justify causal language. [This follows the earlier note that “I'd ask the authors to clarify in their response whether the academic-harm and distress items asked students to directly attribute causation (i.e., "did this issue harm your performance"), since this would explicitly justify the causal phrasing used in the abstract and Results ("the biggest cause of..."). Stating this explicitly in the Methods would preempt likely reader questions on this point.”]*

— I now note this explicitly in the Methods (in the part of the Analyses sub-section where the relevant items are introduced): “For both *performance* and *distress*, the relevant questions in the survey involved direct self-attribution of causation, as in “Within the last 12 months, to what extent did the following issue(s) cause you distress?”. For more information about this, see the SI.” In addition, the reference to the study’s Supplementary Information in the Survey sub-section of the Methods now reads “For more details about the survey, including the specific questions students were asked as part of it, see the Supplementary Information (SI) in the study’s Open Science Framework (OSF) repository…”.

*> 2. Note explicitly that no multiple-comparisons correction was applied, and the rationale (descriptive/exploratory framing).*

­­­— I now note this explicitly in the Analysis sub-section of the Methods, which now says the following (also taking into account reviewer 1’s related comment on effect sizes): “No multiple-comparisons correction was applied, as the related analyses were descriptive and exploratory in nature, with a focus on the results of individual tests rather than a universal null hypothesis, and without placing emphasis on any result in particular in light of the size of its p-value [35,36]. At the same time, associated best-practice guidelines were followed for null hypothesis significance testing, including reporting exact p-values as well as effect sizes. From a practical perspective, however, this did not affect the study’s findings, since p-values for differences were small throughout (generally well below *p* <.001), mainly due to the large samples involved [37,38], which further drove a focus on descriptive analysis of effect sizes in the interpretation of the results.”

*> 3. Distinguish the single-item procrastination measure from validated trait scales used in cited prior literature when drawing comparisons.*

— I now distinguish this through the following addition to the Discussion: “In terms of measurements, it [the survey used in the study] relies on a single-item measure of procrastination, as opposed to the validated scales of procrastination used in most past literature, such as the *Pure Procrastination Scale* and the *Tuckman Procrastination Scale* [24,50].”

*> Data availability: I accessed the OSF repository and confirm it contains the extracted/compiled datasets, the full analysis script, session/environment information, and the Supplementary Information document. This fully satisfies the data-sharing requirement. The only outstanding item is converting the link from a view-only reviewer-access token to a permanent public address prior to final publication.*
— I converted the link from a private reviewer link to a public, permanent address (<https://doi.org/10.17605/OSF.IO/AUYVP>).
