# Supplementary material for: Procrastination among US college students: Prevalence and impact relative to common health and life issues
Source: PLoS One. 2026 Oct 7;21(10):e0357714. doi: 10.1371/journal.pone.0357714 (PMC13645087; doi:10.1371/journal.pone.0357714)
Supplement: Peer review history

# Peer review: Decision Letter 0

**DOI:** 10.1371/journal.pone.0357714.r001

**Authors:** Peter Karl Jonason

7 Aug 2026

Dear Dr. Shatz,

Thank you for submitting your manuscript to PLOS ONE. After careful consideration, we feel that it has merit but does not fully meet PLOS ONE’s publication criteria as it currently stands. Therefore, we invite you to submit a revised version of the manuscript that addresses the points raised during the review process. For instance, consider the issue of potentially overpowered tests leading to conclusions that, while significant, may be practically useless. Consider effect sizes more thoroughly. Please submit your revised manuscript by Oct 05 2026 11:59PM. If you will need more time than this to complete your revisions, please reply to this message or contact the journal office at plosone@plos.org. When you're ready to submit your revision, log on to https://www.editorialmanager.com/pone/ and select the 'Submissions Needing Revision' folder to locate your manuscript file.

- A letter that responds to each point raised by the academic editor and reviewer(s). You should upload this letter as a separate file labeled 'Response to Reviewers'.
- A marked-up copy of your manuscript that highlights changes made to the original version. You should upload this as a separate file labeled 'Revised Manuscript with Track Changes'.
- An unmarked version of your revised paper without tracked changes. You should upload this as a separate file labeled 'Manuscript'.

If you would like to make changes to your financial disclosure, please include your updated statement in your cover letter. Guidelines for resubmitting your figure files are available below the reviewer comments at the end of this letter.

If applicable, we recommend that you deposit your laboratory protocols in protocols.io to enhance the reproducibility of your results. Protocols.io assigns your protocol its own identifier (DOI) so that it can be cited independently in the future. For instructions see: https://journals.plos.org/plosone/s/submission-guidelines#loc-laboratory-protocols. Additionally, PLOS ONE offers an option for publishing peer-reviewed Lab Protocol articles, which describe protocols hosted on protocols.io. Read more information on sharing protocols at https://plos.org/protocols?utm_medium=editorial-email&utm_source=authorletters&utm_campaign=protocols.

As the corresponding author, your ORCID iD is verified in the submission system and will appear in the published article. PLOS supports the use of ORCID, and we encourage all coauthors to register for an ORCID iD and use it as well. Please encourage your coauthors to verify their ORCID iD within the submission system before final acceptance, as unverified ORCID iDs will not appear in the published article. *Only*  the individual author can complete the verification step; PLOS staff *cannot*  verify ORCID iDs on behalf of authors.

We look forward to receiving your revised manuscript.

Kind regards,

Peter Karl Jonason

Academic Editor

PLOS One

Journal Requirements:

When submitting your revision, we need you to address these additional requirements.

1. Please ensure that your manuscript meets PLOS ONE's style requirements, including those for file naming. The PLOS ONE style templates can be found at

https://journals.plos.org/plosone/s/file?id=wjVg/PLOSOne_formatting_sample_main_body.pdf and

https://journals.plos.org/plosone/s/file?id=ba62/PLOSOne_formatting_sample_title_authors_affiliations.pdf

2. If the reviewer comments include a recommendation to cite specific previously published works, please review and evaluate these publications to determine whether they are relevant and should be cited. There is no requirement to cite these works unless the editor has indicated otherwise.

3. Please review your reference list to ensure that it is complete and correct. If you have cited papers that have been retracted, please include the rationale for doing so in the manuscript text, or remove these references and replace them with relevant current references. Any changes to the reference list should be mentioned in the rebuttal letter that accompanies your revised manuscript. If you need to cite a retracted article, indicate the article’s retracted status in the References list and also include a citation and full reference for the retraction notice.

[Note: HTML markup is below. Please do not edit.]

Reviewers' comments:

Reviewer's Responses to Questions

**Comments to the Author**

1. Is the manuscript technically sound, and do the data support the conclusions?

Reviewer #1: Yes

Reviewer #2: Partly

**********

2. Has the statistical analysis been performed appropriately and rigorously?

Reviewer #1: Yes

Reviewer #2: Yes

**********

3. Have the authors made all data underlying the findings in their manuscript fully available?

The PLOS Data policy

Reviewer #1: Yes

Reviewer #2: Yes

**********

4. Is the manuscript presented in an intelligible fashion and written in standard English?

Reviewer #1: Yes

Reviewer #2: Yes

**********

Reviewer #1: This manuscript addresses an important gap in the higher education and behavioral science literature by contextualizing the prevalence, academic impact, and associated distress of procrastination relative to 35 common life and health challenges among college students. Using a large, contemporary, multi-institutional sample from the Spring 2025 ACHA-NCHA survey (N = 65,950), the study demonstrates that procrastination represents a substantial academic concern that rivals or exceeds many commonly recognized student issues. The manuscript is well organized, methodologically transparent, and strengthened by its commitment to reproducibility through the availability of data, analytic code, and study materials.

Major Strengths

The use of a large, nationwide, multi-institution dataset provides substantially greater generalizability than prior studies that have relied primarily on single-institution or smaller convenience samples.

Comparing procrastination with 35 other life and health concerns offers valuable context for understanding its relative prevalence and impact, an approach that is largely absent from the existing literature.

The distinction between full-sample and conditional-impact analyses is particularly informative and effectively demonstrates why procrastination constitutes a population-wide challenge, reflecting both its high prevalence and considerable academic consequences.

Separate analyses for undergraduate and graduate/professional students reveal meaningful differences across educational levels and add important nuance to the findings.

The availability of data sources, R scripts, and methodological documentation enhances transparency, reproducibility, and opportunities for future research.

Minor Concerns and Suggestions

The manuscript states that procrastination and stress are "functionally equal in prevalence" despite a statistically significant difference (72.3% vs. 73.8%, p < .001). Given the large sample size, statistical significance may not necessarily indicate practical significance. The authors should either justify the use of the term "functionally equal" by discussing practical equivalence or rephrase the statement as "similar in prevalence" and consider reporting an effect size.

One of the most interesting findings is the discrepancy between procrastination's ranking in overall distress and its lower ranking in conditional distress. Expanding the discussion of why procrastination imposes such a large population-level burden despite not producing the highest conditional distress would strengthen the practical implications for intervention prioritization.

Although the manuscript acknowledges potential bidirectional relationships between procrastination and factors such as stress, anxiety, and sleep difficulties, a more explicit discussion of how these overlapping and reciprocal relationships may influence interpretation of the comparative findings would add theoretical depth.

The discussion suggests that lower rates of procrastination among graduate and professional students may reflect age-related maturity or selection effects. It may also be worthwhile to consider whether structural differences in graduate education, such as greater autonomy and fewer short-term assignment deadlines, contribute to these observed differences.

Figures are currently placed after the reference section. While this is acceptable for review purposes, they should be appropriately integrated or clearly indicated in the final publication format.

Additional Comments

The manuscript appropriately addresses ethical considerations, including informed consent, institutional review approvals, and data anonymization procedures. No ethical concerns were identified. The study appears to represent original work, and the use of publicly available ACHA-NCHA data is properly acknowledged and cited.

Overall Recommendation

This is a strong, timely, and methodologically rigorous manuscript that makes a meaningful contribution to the literature on student procrastination. The suggested revisions are relatively minor and are intended primarily to improve the interpretation and discussion of the findings.

Reviewer #2: This manuscript presents a large-scale, descriptive analysis of procrastination prevalence and impact using Spring 2025 ACHA-NCHA data (N = 65,950 students, 97 institutions), comparing it against 35 other student life and health issues. The study addresses a genuine gap in the literature, which has relied heavily on small, dated samples (e.g., Potts, 1987, N = 95), and the scale and recency of this dataset is a meaningful contribution to the field.

Technical soundness and statistical rigor: The statistical methods are appropriate and rigorously applied. Chi-squared tests of proportion are a correct choice for the comparisons made, and the use of Wilson-method confidence intervals is methodologically sound, particularly for proportions near 0% or 100% (e.g., hazing, ~1%). The authors appropriately recognize that with N = 65,950, p-values alone are uninformative, and correctly pivot to ratios and rank comparisons as the substantively meaningful metrics (e.g., explicitly calling stress and procrastination "functionally equal" in prevalence despite p < .001). This shows good statistical judgment.

Conclusions and data support: The core conclusions follow appropriately from the data presented. I'd ask the authors to clarify in their response whether the academic-harm and distress items asked students to directly attribute causation (i.e., "did this issue harm your performance"), since this would explicitly justify the causal phrasing used in the abstract and Results ("the biggest cause of..."). Stating this explicitly in the Methods would preempt likely reader questions on this point.

Data availability: I accessed the OSF repository and confirm it contains the extracted/compiled datasets, the full analysis script, session/environment information, and the Supplementary Information document. This fully satisfies the data-sharing requirement. The only outstanding item is converting the link from a view-only reviewer-access token to a permanent public address prior to final publication.

Suggestions for revision:

1. Clarify whether harm/distress items involve direct self-attribution of causation, to justify causal language.

2. Note explicitly that no multiple-comparisons correction was applied, and the rationale (descriptive/exploratory framing).

3. Distinguish the single-item procrastination measure from validated trait scales used in cited prior literature when drawing comparisons.

No concerns regarding research ethics, dual publication, or publication ethics. I recommend minor revision.

**********

what does this mean?). If published, this will include your full peer review and any attached files.

If you choose “no”, your identity will remain anonymous but your review may still be made public.

**Do you want your identity to be public for this peer review?**  For information about this choice, including consent withdrawal, please see our Privacy Policy

Reviewer #1: No

Reviewer #2: **Yes:** Anam Fatima

**********

[NOTE: If reviewer comments were submitted as an attachment file, they will be attached to this email and accessible via the submission site. Please log into your account, locate the manuscript record, and check for the action link "View Attachments". If this link does not appear, there are no attachment files.]

To ensure your figures meet our technical requirements, please review our figure guidelines: https://journals.plos.org/plosone/s/figures

You may also use PLOS’s free figure tool, NAAS, to help you prepare publication quality figures: https://journals.plos.org/plosone/s/figures#loc-tools-for-figure-preparation.

NAAS will assess whether your figures meet our technical requirements by comparing each figure against our figure specifications.

# Author comment: Author response to Decision Letter 1

**DOI:** 10.1371/journal.pone.0357714.r002

19 Aug 2026

Dear editor and reviewers,

Thank you for the thoughtful comments and for the opportunity to revise the manuscript! I appreciate them both.

Below, I explain how I revised the manuscript to address your comments. For concision and clarity, I included only comments with suggestions for the manuscript (e.g., as opposed to ones summarizing it or discussing its strengths).

REVIEWER 1

> The manuscript states that procrastination and stress are "functionally equal in prevalence" despite a statistically significant difference (72.3% vs. 73.8%, p < .001). Given the large sample size, statistical significance may not necessarily indicate practical significance. The authors should either justify the use of the term "functionally equal" by discussing practical equivalence or rephrase the statement as "similar in prevalence" and consider reporting an effect size.

— I added the following clarification: “Note that here and elsewhere in the paper, the term “functionally equal” is used when the ratio between the prevalence or impact of procrastination and another issue was close enough to a null value to be reported as 1.0 (as in the case of the prevalence of procrastination and stress, where the ratio_prevalence was 0.723/0.738 ≈ 1.0). This focus on effect size when there is high similarity between the two values is particularly important given this study’s large sample, since “overpowered” tests often indicate even tiny differences as statistically significant [37,38].”

In addition, the impact of large samples on p-values and the implications of this for the analyses are now mentioned explicitly in the Methods (see the response to comment #2 by reviewer 2). All these edits also aim to take into account the editor’s related comment (“For instance, consider the issue of potentially overpowered tests leading to conclusions that, while significant, may be practically useless.”) and the relevant assessment by reviewer 2 in their original review (“The authors appropriately recognize that with N = 65,950, p-values alone are uninformative, and correctly pivot to ratios and rank comparisons as the substantively meaningful metrics (e.g., explicitly calling stress and procrastination "functionally equal" in prevalence despite p < .001). This shows good statistical judgment.”).

> One of the most interesting findings is the discrepancy between procrastination's ranking in overall distress and its lower ranking in conditional distress. Expanding the discussion of why procrastination imposes such a large population-level burden despite not producing the highest conditional distress would strengthen the practical implications for intervention prioritization.

— I have expanded the discussion on this as suggested. The relevant part now reads:

“First, although procrastination had the biggest impact in the full sample (among all students), other issues sometimes had bigger impact in conditional terms (among students who suffered from any given issue), especially when it comes to graduate & professional students and to distress. The reason for this apparent discrepancy is that even in cases where procrastination did not have the highest conditional impact, its conditional impact was still high enough that, when combined with its prevalence, its total impact ranked at or near the top of all issues, indicating a high relative burden within each population. This highlights how differences in the total impact of issues across populations—in this case of procrastination among undergraduate students compared to graduate & professional ones—are attributable to differences in both the prevalence of each issue and its conditional impact. This is also an important reminder that even if a certain issue only affects a small subset of students, its impact can be significant for those students. A key example of this is ADHD: despite having similar conditional impact on students’ academic performance as procrastination (64.1% compared to 62.7%), it affected “only” 12.9% of students compared to procrastination’s 45.4%, because only 20.2% of students experienced it compared to procrastination’s 72.3% (as shown in Fig 4).”

> Although the manuscript acknowledges potential bidirectional relationships between procrastination and factors such as stress, anxiety, and sleep difficulties, a more explicit discussion of how these overlapping and reciprocal relationships may influence interpretation of the comparative findings would add theoretical depth.

— Added an explicit discussion on this:

“For another, there are various potential relationships between the different issues, which could influence the impact of any given issue. Stress, for instance, was a top issue, but procrastination was a top cause of distress, and procrastination itself can be caused or exacerbated by this and other issues, like anxiety and lack of sleep, potentially—but not necessarily—in a bidirectional manner that results in vicious cycles [1,46–49]. An example of such a feedback loop is a student procrastinating on a task, which causes them to feel stressed, which interferes with their sleep, which causes them to procrastinate on the task further.

These potential interplays add an element of uncertainty when comparing the impact of the different issues that students face, since it is unclear to what extent students’ reporting accounts for such higher-order effects. Notably, it is possible that any given issue has greater impact on students than it initially appears to, because it is the upstream cause of downstream issues that are then reported to hurt students’ academic performance or cause them distress. For example, it is possible that a portion of the negative effect of students’ stress on their academic performance should be attributed to procrastination, and that, at the same time, a portion of procrastination’s own negative effect should be partially attributed to ADHD.

From a practical perspective, the key concern is that we might under- or over-estimate the impact of different issues, and mainly that we will prioritize issues that are symptoms of other issues, in cases where treating the underlying cause is more important. To address these uncertainties, it would help to conduct further research into when and to what extent these factors cause and are caused by procrastination, and to what degree these higher-order effects are reflected in students’ reporting. Nevertheless, although these uncertainties necessitate added caution when interpreting the impact of the issues that were examined in this study, the present findings represent at least first-order approximations of impact, which are a useful starting point for current prioritization and for any future work.”

> The discussion suggests that lower rates of procrastination among graduate and professional students may reflect age-related maturity or selection effects. It may also be worthwhile to consider whether structural differences in graduate education, such as greater autonomy and fewer short-term assignment deadlines, contribute to these observed differences.

— I updated the discussion to reflect that these and additional structural factors could play a role in the difference in procrastination rates across the two student groups. The relevant part now reads: “The prominence of these negative effects applied both to undergraduate students and to graduate & professional ones, although undergraduates consistently suffered more from procrastination. This could be attributable to various causes, such as: age differences, with younger people procrastinating more on average [3,7]; selection effects, in terms of which students proceed to advanced degrees; and structural differences across educational programs, when it comes to factors like the level of autonomy given to students, the degree of external accountability students have, the expected standards for students’ performance, and the types of academic tasks and deadlines that students are assigned.”

> Figures are currently placed after the reference section. While this is acceptable for review purposes, they should be appropriately integrated or clearly indicated in the final publication format.

— The location of the figures is now clearly indicated in the body via their captions per PLOS’s requirements, with the final placement and integration of the figure files into the manuscript confirmed to be handled by PLOS’s production team before publication.

REVIEWER 2

> 1. Clarify whether harm/distress items involve direct self-attribution of causation, to justify causal language. [This follows the earlier note that “I'd ask the authors to clarify in their response whether the academic-harm and distress items asked students to directly attribute causation (i.e., "did this issue harm your performance"), since this would explicitly justify the causal phrasing used in the abstract and Results ("the biggest cause of..."). Stating this explicitly in the Methods would preempt likely reader questions on this point.”]

— I now note this explicitly in the Methods (in the part of the Analyses sub-section where the relevant items are introduced): “For both performance and distress, the relevant questions in the survey involved direct self-attribution of causation, as in “Within the last 12 months, to what extent did the following issue(s) cause you distress?”. For more information about this, see the SI.” In addition, the reference to the study’s Supplementary Information in the Survey sub-section of the Methods now reads “For more details about the survey, including the specific questions students were asked as part of it, see the Supplementary Information (SI) in the study’s Open Science Framework (OSF) repository…”.

> 2. Note explicitly that no multiple-comparisons correction was applied, and the rationale (descriptive/exploratory framing).

— I now note this explicitly in the Analysis sub-section of the Methods, which now says the following (also taking into account reviewer 1’s related comment on effect sizes): “No multiple-comparisons correction was applied, as the related analyses were descriptive and exploratory in nature, with a focus on the results of individual tests rather than a universal null hypothesis, and without placing emphasis on any result in particular in light of the size of its p-value [35,36]. At the same time, associated best-practice guidelines were followed for null hypothesis significance testing, including reporting exact p-values as well as effect sizes. From a practical perspective, however, this did not affect the study’s findings, since p-values for differences were small throughout (generally well below p <.001), mainly due to the large samples involved [37,38], which further drove a focus on descriptive analysis of effect sizes in the interpretation of the results.”

> 3. Distinguish the single-item procrastination measure from validated trait scales used in cited prior literature when drawing comparisons.

— I now distinguish this through the following addition to the Discussion: “In terms of measurements, it [the survey used in the study] relies on a single-item measure of procrastination, as opposed to the validated scales of procrastination used in most past literature, such as the Pure Procrastination Scale and the Tuckman Procrastination Scale [24,50].”

> Data availability: I accessed the OSF repository and confirm it contains the extracted/compiled datasets, the full analysis script, session/environment information, and the Supplementary Information document. This fully satisfies the data-sharing requirement. The only outstanding item is converting the link from a view-only reviewer-access token to a permanent public address prior to final publication.

— I converted the link from a private reviewer link to a public, permanent address (https://doi.org/10.17605/OSF.IO/AUYVP).

Attachment Submitted filename: Response to Reviewers.docx

# Editor report: Decision Letter 1

**DOI:** 10.1371/journal.pone.0357714.r003

**Authors:** Peter Karl Jonason

22 Aug 2026

Procrastination among US college students: Prevalence and impact relative to common health and life issues

PONE-D-26-21890R1

Dear Dr. Shatz,

We’re pleased to inform you that your manuscript has been judged scientifically suitable for publication and will be formally accepted for publication once it meets all outstanding technical requirements.

Within one week, you’ll receive an e-mail detailing the required amendments. When these have been addressed, you’ll receive a formal acceptance letter and your manuscript will be scheduled for publication.

An invoice will be generated when your article is formally accepted. Please note, if your institution has a publishing partnership with PLOS and your article meets the relevant criteria, all or part of your publication costs will be covered. Please make sure your user information is up-to-date by logging into Editorial Manager at Editorial Manager® and clicking the ‘Update My Information' link at the top of the page. For questions related to billing, please contact billing support.

If your institution or institutions have a press office, please notify them about your upcoming paper to help maximize its impact. If they’ll be preparing press materials, please inform our press team as soon as possible -- no later than 48 hours after receiving the formal acceptance. Your manuscript will remain under strict press embargo until 2 pm Eastern Time on the date of publication. For more information, please contact onepress@plos.org.

Kind regards,

Peter Karl Jonason

Academic Editor

PLOS One

Additional Editor Comments (optional):

Reviewers' comments:

# Editor report: Acceptance letter

**DOI:** 10.1371/journal.pone.0357714.r004

**Authors:** Peter Karl Jonason

PONE-D-26-21890R1

PLOS One

Dear Dr. Shatz,

I'm pleased to inform you that your manuscript has been deemed suitable for publication in PLOS One. Congratulations! Your manuscript is now being handed over to our production team.

At this stage, our production department will prepare your paper for publication. This includes ensuring the following:

* All references, tables, and figures are properly cited

* All relevant supporting information is included in the manuscript submission,

* There are no issues that prevent the paper from being properly typeset

You will receive further instructions from the production team, including instructions on how to review your proof when it is ready. Please keep in mind that we are working through a large volume of accepted articles, so please give us a few days to review your paper and let you know the next and final steps.

Lastly, if your institution or institutions have a press office, please let them know about your upcoming paper now to help maximize its impact. If they'll be preparing press materials, please inform our press team within the next 48 hours. Your manuscript will remain under strict press embargo until 2 pm Eastern Time on the date of publication. For more information, please contact onepress@plos.org.

You will receive an invoice from PLOS for your publication fee after your manuscript has reached the completed accept phase. If you receive an email requesting payment before acceptance or for any other service, this may be a phishing scheme. Learn how to identify phishing emails and protect your accounts at https://explore.plos.org/phishing.

If we can help with anything else, please email us at customercare@plos.org.

Thank you for submitting your work to PLOS One and supporting open access.

Kind regards,

PLOS One Editorial Office Staff

on behalf of

Dr. Peter Karl Jonason

Academic Editor

PLOS One
